# The progression of non-culprit coronary lesion is related to higher SII, SIRI, and PIV in patients with ACS

**DOI:** 10.1097/MD.0000000000041094

**Published:** 2024-12-27

**Authors:** Zhou Yilu, Wang Zhanglong, Huang Fanke, Guan Jing, Wang Yue, Chen Yuwen, Li Bingqing, Lv Jianfeng

**Affiliations:** a Medical College, China Three Gorges University, Yichang, Hubei, China; b Department of Cardiology, Renhe Hospital Affiliated to Three Gorges University, Yichang, Hubei, China.

**Keywords:** acute coronary syndrome, non-culprit coronary lesion, pan-immune-inflammation value, percutaneous coronary intervention, progression, system inflammation response index, systemic immune inflammation index

## Abstract

Coronary heart disease pathogenesis is intricately linked to inflammation. Acute coronary syndrome (ACS) is a coronary heart disease that seriously affects the prognosis of patients. New immune-inflammatory indices such as systemic immune inflammation index (SII), system inflammation response index (SIRI), and pan-immune-inflammation value (PIV) have emerged as potential biomarkers, offering reflection into systemic inflammatory states and assisting in the prognosis of diverse diseases. This research explored the association between the new immune-inflammatory indices (SII, SIRI, and PIV) and the progression of non-culprit coronary lesions (NCL) in patients with ACS after percutaneous coronary intervention (PCI). Our study investigated the potential association between the immune-inflammatory index (SII, SIRI, and PIV) and NCL progression in patients with ACS following PCI. We conducted a retrospective analysis of patients with ACS who underwent PCI twice at a single-center from 2019 to 2023. Clinical and angiographic features were collected from electronic medical records. The primary outcome was NCL progression. All patients were divided into a progression group and a non-progression group based on angiographies. The clinical and angiographic features were analyzed. The study included 311 ACS patients (progression group: 97 males, 34 females; non-progression group: male 146 males, 34 females). The SII, SIRI, and PIV were significantly higher in the NCL progression group than in the non-progression group (*P* < .001). Logistic regression analysis showed that SII, SIRI, and PIV were independent risk factors for the NCL progression and positively correlated with it (OR: 1.002, *P* < .001; OR: 2.188, *P* < .001; OR: 1.003, *P* < .001). ROC showed that the SII value was the highest in terms of sensitivity with a value of 67.18% (AUC = 0.7288, *P* < .001), and the SIRI was the highest in terms of specificity with a value of 79.44% (AUC = 0.6974, *P* < .001). The SII, SIRI, and PIV are valuable predictors of NCL progression in patients with ACS. Higher SII, SIRI, and PIV are related to the progression of NCL.

## 1. Introduction

Cardiovascular diseases are the leading causes of death worldwide.^[1]^ Acute coronary syndrome (ACS) is a group of syndromes of acute myocardial ischemia and necrosis caused by partial or complete stenosis and occlusion of the coronary artery lumen after atherosclerotic plaque rupture or thrombosis.^[2]^ Although percuraneous coronary intervention (PCI)can restore myocardial blood perfusion and relieve symptoms in the shortest time, it is aimed at treating culprit lesions and ignores the treatment of non-culprit coronary lesion (NCL). Previous studies have shown that major adverse cardiovascular events (MACE) after PCI involve the progression of NCL.^[3]^ Different from PCI only for culprit coronary lesion (CL), PCI for NCL can significantly reduce the poor prognosis when it achieves complete revascularization.^[4]^ The rapid progress of NCL after PCI may be one of the reasons for the decrease of PCI benefit.^[5]^ Many studies show that vulnerable plaques in ACS patients exist not only in CL but also in NCL.^[6,7]^ When occurs ACS or after PCI, the body has a rapid increase in the inflammatory response, which may be closely related to the progress of NCL.

Recently, there has been a concern about the role of low-grade inflammatory injury in the disease process. Some scholars believe that patients with systemic inflammatory diseases have a higher risk of cardiovascular diseases.^[8]^ The role of immune-inflammatory system activation in the development of atherosclerotic plaques has been assumed. Based on previous studies, the Immune-inflammatory system causes endothelial dysfunction through oxidative modification, phagocytosis, adhesion migration, and thrombosis, which plays an important role in the development of atherosclerosis.^[9–14]^ Therefore, the markers of immune inflammation have become a research hotspot. The number of inflammatory cells in the blood routine and its derived indexes are the core factors of inflammatory reaction. A series of new indicators, such as SII, SIRI, and PIV have been studied in various systems in recent years, especially in tumor and surgical patients, because of their simplicity and availability. However, the correlation between the new immune-inflammatory markers SII, SIRI, and PIV in the progression of noncriminal vascular diseases after PCI has not been fully studied.

In a retrospective cohort of ACS patients who have undergone PCI, we aimed to explore the relationship between new immune-inflammatory indexes SII, SIRI, and PIV and the progress of NCL, and to explore the value of these indexes in predicting the progress of NCL.

## 2. Methods

### 2.1. Patient selection

This retrospective study conformed to the Declaration of Helsinki and was approved by the Ethics Committee in the Renhe Affiliated Hospital of China Three Gorges University. All patients underwent PCI as a treatment for the CL.

Patients with coronary heart disease (CHD) who previously received PCI in the cardiovascular medicine of our hospital from January 2019 to December 2023 were retrospectively studied. The inclusion criteria were: age ≥ 18 years old; All patients were given aspirin, clopidogrel, or tigrellol before the operation; and there were no contraindications for coronary angiography (CAG), and carotid ultrasound. The exclusion criteria were as follows: Patients with severe heart failure (NYHA III or IV), severe myocarditis, and congenital heart disease; Life-threatening patients with tumors, infectious diseases, or severe liver and kidney diseases; For the first time, CAG indicated that all 3 main coronary arteries were completely occluded; the history of coronary artery bypass grafting surgery; and incomplete clinical and laboratory data. Finally, a total of 331 patients were enrolled in this study.

### 2.2. Data collection and definition

All patients` clinical data and laboratory were obtained from hospital medical records. It includes sociodemographic characteristics, medical history, and laboratory results derived from hospital medical records. Blood samples were measured on the day of admission or the next day, encompassing fasting blood glucose (FBG), neutrophils, monocytes, lymphocytes, platelets, creatinine, uric acid (UA), total cholesterol (TC), triglycerides (TG), high-density lipoprotein cholesterol (HDL-C), and low-density lipoprotein cholesterol (LDL-C).

### 2.3. Angiogram and analysis

CAG imaging analysis and the definitions of progression in NCL. All patients underwent multi-position projection. The angiographic images were independently analyzed by 2 cardiologists with more than ten years of experience unaware of the patient’s clinical information. The vessels treated by PCI were defined as culprit lesions. All patients underwent PCI for the culprit lesions. Those vessels that were not associated with ischemic symptoms or positive functional ischemia tests and did not require PCI at baseline were defined as NCL.

Definition of NCL progression^[15]^: The stenosis degree of the NCL was ≥ 50% at the time of baseline PCI, and the degree of NCL progression was ≥ 10% at the time of angiographic follow-up; The stenosis degree of the NCL was < 50% at the time of baseline PCI, and the degree of NCL progression was ≥ 30% at the time of angiographic follow-up; The degree of NCL progression ≥ 30%, while there were no NCL at the time of baseline PCI; and NCL progression to total occlusion.

The NCL progression group was further divided into mild (n = 85), moderate (n = 25), and severe progressive groups (n = 21) according to the degree of progress. The degree of stenosis increased by <50%, 50% to 70%, and more than 70% respectively. When multiple NCL progressions were found, the lesions with the most significant increase in stenosis grade in the follow-up were taken as the index lesions.

**SII** was calculated as (neutrophil count [10^9^/L]) · (platelet count [10^9^/L])/ (lymphocyte count [10^9^/L]);

**SIRI** was (neutrophil count [10^9^/L]) · (monocyte count [10^9^/L])/ (lymphocyte count [10^9^/L]);

**PIV** was calculated as (neutrophil count [10^9^/L]) · (platelet count [10^9^/L]) · (monocyte count [10^9^/L])/(lymphocyte count [10^9^/L]).^[16]^

The severity of coronary lesions was quantitatively evaluated by the Gensini score assessment system and scored by 2 independent senior cardiologists. The degree of stenosis and the coronary artery lesion site were scored as follows: 1 point for ≤ 25% narrowing, 2 points for 26% to 50% narrowing, 4 points for 51% to 75% narrowing, 8 points for 76% to 90% narrowing, 16 points for 91% to 99% narrowing, and 32 points for total occlusion. After that, each lesion score is multiplied by a factor considering the importance of the lesion’s position in the coronary circulation. The final integral, which represents the degree of coronary artery disease of each patient, is obtained by summing the integrals of each branch.^[17]^

### 2.4. Statistical analysis

All statistical analyses were performed using Prism 9.0 (GraphPad) and SPSS v26.0 (IBM Corp.). Continuous variables were examined by the Kolmogorov–Smirnov test. Continuous variables are expressed as mean ± SD and categorical data are presented as percentages. Differences in continuous variables between the 2 groups were assessed by unpaired 2-tailed *t*-test and the Mann–Whitney *U*-test, as appropriate. Categorical data and proportions were analyzed by chi-square test. Correlations between the inflammation indies (SII, SIRI, and PIV) and the Gensini score were analyzed with bivariate correlation. Binary logistic regression analysis was performed to examine independent risk factors for the progression of NCL. Receiver operating characteristic (ROC) analysis and a calculation of sensitivity and specificity were performed to test the ability of indices to predict the progression of NCL. For the multivariate regression analysis, the parameters with a *P* < .1 in the univariate analysis were included in the model. The effects of different variables in the progressive group were calculated with univariate analysis. 2-tailed *P* < .05 was considered statistically significant.

**Figure 1.**
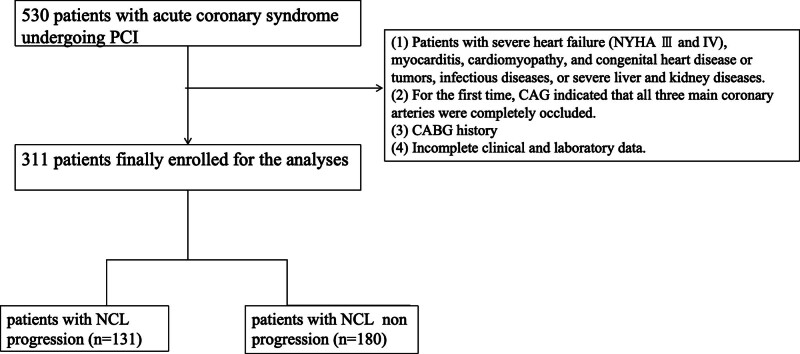
Patients with CHD who previously received PCI in the cardiovascular medicine of our hospital from January 2019 to December 2023 were retrospectively studied. The inclusion criteria were age ≥ 18 years old; all patients were given aspirin, clopidogrel, or tigrellol before the operation; there were no contraindications for CAG, and carotid ultrasound. The exclusion criteria were as follows: Patients with severe heart failure (NYHA III or IV), severe myocarditis, and congenital heart disease. Life-threatening patients with tumors, infectious diseases, or severe liver and kidney diseases. For the first time, CAG indicated that all 3 main coronary arteries were completely occluded. The history of coronary artery bypass grafting surgery. Incomplete clinical and laboratory data. Finally, a total of 331 patients were enrolled in this study. CAG = coronary angiography, CHD = coronary heart disease, NYHA = New York Heart Association, PCI = percutaneous coronary intervention.

311 patients were divided into 2 groups: the progression group (n = 131) and the non-progression group (n = 180) by the definition of NCL progression (Fig. 1).

## 3. Results

### 3.1. Baseline clinical characteristics

Of the 331 patients, the progression group was observed in 131 (42.12%). Patients in the progression group were older (65.0 > 61.5, *P* = .043) and more smoking (63.4%>50.6%, *P* = .025). Men were more in both groups (74%, 81%). No significant difference existed in previous history of hypertension, diabetes, and ischemic stroke. The use of statins is more in the nonprogressive group (*P* < .001). The baseline clinical characteristics of the patients are shown in Table 1

**Table 1:** Demographics and clinical characteristics of the patient.

Variable	Progression (n = 131)	Non-progression (n = 180)	*t/χ* ^2^	*P*
Men gender (%)	97 (74.0)	146 (81.1)		.137
Age (%)	65.0 (56.0, 70.0)	61.5 (53.0, 69.0)	−2.020	**.043**
BMI (kg/m^2^)	24.60 ± 0.31	23.55 ± 0.24	−2.702	**.007**
Hypertension (%)	91 (69.5)	116 (64.4)		.354
Hyperlipidemia (%)	22 (16.8)	36 (20.0)		.474
Diabetes mellitus (%)	48 (36.6)	50 (27.8)	2.760	**.097**
Ischemic stroke (%)	51 (38.9)	54 (30.0)		.100
Smoking (%)	83 (63.4)	91 (50.6)	5.043	**.025**
Drinking (%)	54 (41.2)	82 (45.6)		.447
Aspirin (%)	12 (94.7)	176 (97.8)		.141
Beta-blockers (%)	73 (55.7)	110 (61.1)		.341
Calcium antagonists (%)	43 (32.8)	67 (37.2)		.423
Nitrates (%)	13 (9.9)	22 (12.2)		.527
ACEI/ARB (%)	59 (45.7)	83 (46.1)		.851
Statins (%)	110 (84.0)	174 (96.7)	15.418	**<.001**

ACEI/ARB = angiotensin-converting enzyme inhibitor/angiotensin receptor blocker, BMI = body mass index.

**Figure 2.**
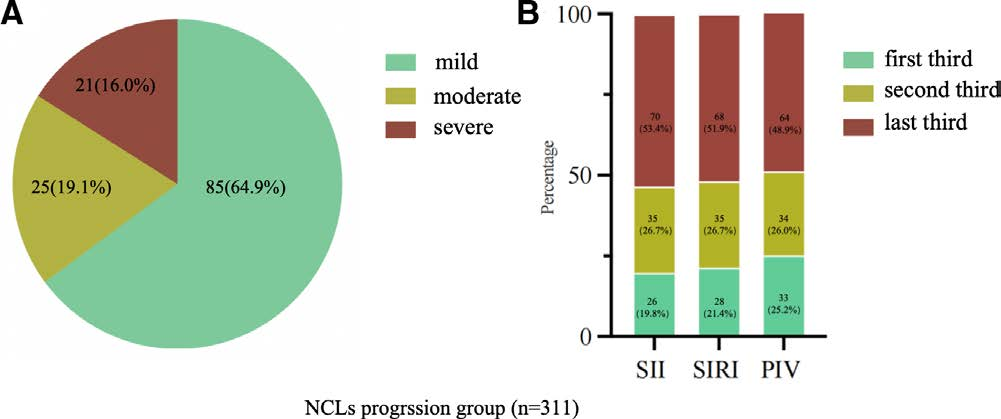
In the progression group, there were 85 (64.9%) patients with mild to moderate stenosis, 25 (19.1%) patients with mild to severe stenosis, and 21 (16.0%) patients with moderate to severe stenosis. Among the tertiles of SII, the progress rate is 19.8%, 26.7%, and 53.4%. For SIRI, the progress rate is 21.4%, 26.7%, and 51.9%. About PIV, the progress rate is 25.2%, 26.0%, and 48.9%. PIV = pan-immune-inflammation value, SII = systemic immune inflammation index, SIRI = system inflammation response index.

Significant differences in laboratory results were observed between the progression group and the non-progression group. The progression group exhibited elevated neutrophils, FBG, TG, and LDL-C levels, while lymphocytes and HDL-C levels were significantly lower compared to the non-progression group (*P* < .05). No significant differences were observed in other laboratory findings such as monocytes, platelets, creatinine, UA, and TC. SII, SIRI, and PIV were significantly higher in the progressive group (all *P* < .05) (Table 2). In the progression group, there were 85 (64.9%) patients with mild to moderate stenosis, 25 (19.1%) patients with mild to severe stenosis, and 21 (16.0%) patients with moderate to severe stenosis. Among the tertiles of SII, the progress rate is 19.8%, 26.7%, and 53.4%. For SIRI, the progress rate is 21.4%, 26.7%, and 51.9%. About PIV, the progress rate is 25.2%, 26.0%, and 48.9% (Fig. 2).

**Table 2:** Laboratory characteristics.

Variable	Progression (n = 131)	Non-progression (n = 180)	*z*/χ^2^	*P*
Neutrophils (10^9^/L)	4.37 (3.57, 5.42)	3.48 (2.82, 4.23)	−5.594	<.001
Monocytes (10^9^/L)	0.38 (0.30, 0.48)	0.38 (0.29, 0.47)		.974
Lymphocytes (10^9^/L)	1.10 (0.92, 1.41)	1.40 (22.2, 26.4)	−6.023	<.001
Platelet (10^9^/L)	182.56 ± 53.55	176.50 ± 51.29		.426
Creatinine (μmol/L)	77.2 (63.6, 90.3)	75.7 (65.3, 88.7)		.917
Uric acid (μmol/L)	359.39 ± 111.47	347.35 ± 90.87		.311
FBG (mmol/L)	5.90 (5.27, 7.18)	5.51 (5.16, 6.20)	−3.028	.002
TC (mmol/L)	3.55 (3.01, 4.53)	3.52 (3.06, 4.08)		.176
TG (mmol/L)	1.33 (0.96, 2.06)	1.16 (0.84, 1.49)	−3.245	.001
HDL-C (mmol/L)	1.10 (0.97, 1.32)	1.20 (1.03, 1.47)	−2.547	.011
LDL-C(mmol/L)	1.73 (1.34, 2.27)	1.62 (1.28, 1.97)	−2.356	.018
SII	668.22 (439.48, 1052.47)	435.55 (289.76, 562.45)	−6.89	<.001
SIRI	1.47 (0.94, 2.24)	0.92 (0.60, 1.32)	−5.934	<.001
PIV	259.99 (139.84, 430.39)	161.44 (96.44, 248.23)	−5.298	<.001

FBG = fasting blood glucose, HDL-C = high-density lipoprotein cholesterol, LDL-C = low-density lipoprotein cholesterol, PIV = pan-immune-inflammation value, SII = systemic immune inflammation index, SIRI = system inflammation response index, TC = total cholesterol, TG = triglyceride.

**Figure 3.**
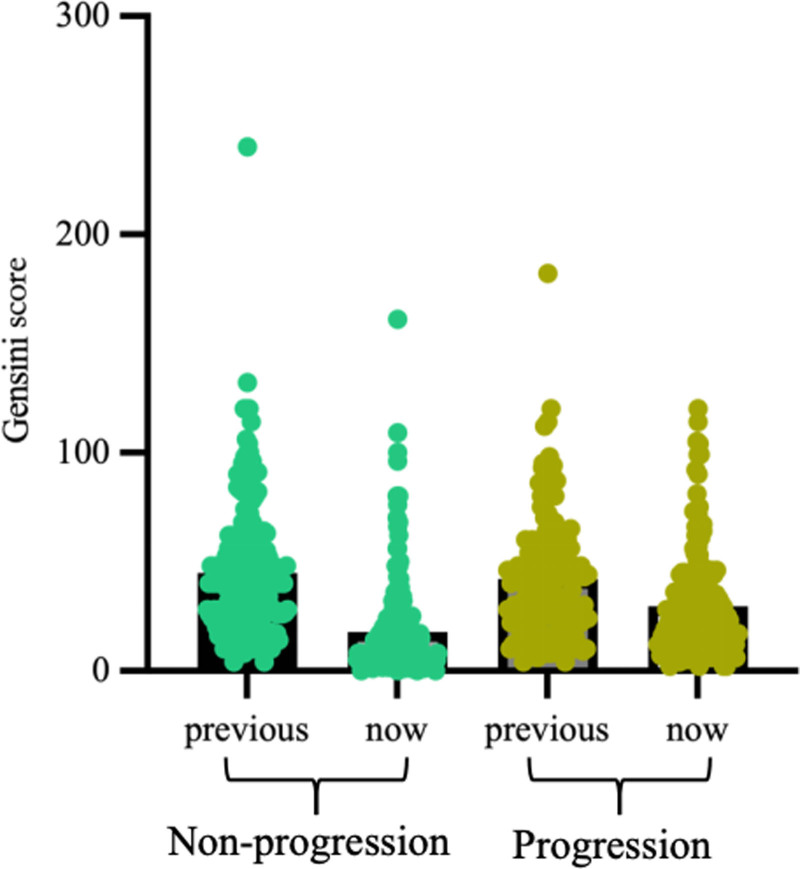
Gensini score 2 was significantly higher in the progressive group (25.0 > 10.0, *P* < .001).

The median interval between 2 angiograms in the progressive group and nonprogressive group is respectively 384 and 374 days. Gensini score 2 was significantly higher in the progressive group (25.0 > 10.0, *P* < .001) (Fig. 3). ΔGS in the progressive group was less than that in the nonprogressive group (16.0 < 22.0, *P* = .002). Left anterior descending lesions were common in the 2 groups (96.2%, 88.3%), especially in the progression group (*P* = .014). The rate of restenosis was not different between patients with progression and those without (Table 3).

**Table 3:** Angiographic findings.

Variable	Progression (n = 131)	Non-progression (n = 180)	*z/χ* ^2^	*P*
Intervals between CAG, d	374.0 (259.0, 802.0)	374.0 (288.0, 490.8)		.107
Vascular lesions (%)				
Single	61 (46.6)	94 (52.2)		.325
Multi	70 (53.4)	86 (47.8)		
Lesion site, n (%)				
LAD	126 (96.2)	159 (88.3)	−2.467	**.014**
LCX	58 (38.2)	71 (41.1)		.393
RCA	46 (35.1)	72 (37.2)		.703
Gensini score1	42.0 (24.0, 58.0)	40.0 (24.0, 59.0)		.994
Gensini score2	25.0 (12.5, 40.0)	10.0 (5.0, 22.0)	−6.852	**<.001**
ΔGS	16.0 (7.5, 33.0)	22.0 (11.0, 41.5)	−3.143	.002
Restenosis (%)	41 (31.3)	46 (25.6)		.265

CAG = coronary angiography, Gensini score1 = Gensini score after primary PCI, Gensini score2 = Gensini score after second PCI, LAD = left anterior descending coronary artery, LCX = left circumflex artery, RCA = right coronary artery, PCI = percutaneous coronary intervention, ΔGS = Gensini score1-Gensini score2 .

### 3.2. Multivariate logistic regression analysis

The independent determinants of NCL were identified using univariate and multivariate logistic regression analyses. In the univariate analysis, variables such as age, BMI, smoking, Gensini score2, TG, TC, HDL, LDL, FBG, SII, SIRI, and PIV were associated with the NCL. The multivariate logistic regression analysis revealed that age, smoke, SII.SIRI and PIV were independent predictors of the NCL (Table 4).

**Table 4:** Multivariate analysis of risk factors for lesion progression.

Variable	Univariate analysis			Multivariate analysis		
	OR	95% CI	*P*	OR	95% CI	*P*
Age	1.027	1.003, 1.052	.029	1.043	1.013, 1.073	.004
BMI	1.097	1.025, 1.176	.008	1.064	0.979, 1.156	.147
DM	1.504	0.928, 2.436	.097	1.194	0.648, 2.200	.569
Smoke	1.691	1.067, 2.679	.025	1.844	1.073, 3.169	.027
TC	1.349	1.066, 1.706	.013	0.877	0.445, 1.728	.705
TG	1.724	1.244, 2.390	.001	1.484	1.000, 2.203	.05
HDL-c	0.434	0.207, 0.912	.028	0.694	0.230, 2.096	.518
LDL-c	1.829	1.290, 2.594	.001	1.964	0.818, 4.712	.131
FBG	1.194	1.055, 1.352	.005	1.081	0.937, 1.247	.286
SII*	1.002	1.002, 1.003	<.001	1.002	1.001, 1.003	<.001
SIRI*	2.238	1.653, 3.030	<.001	2.188	1.587, 3.018	<.001
PIV*	1.003	1.002, 1.005	<.001	1.003	1.002, 1.005	<.001

BMI = body mass index, DM = diabetes mellitus, FBG = fasting blood-glucose, HDL-c = high-density lipoprotein cholesterol, LDL-c = low-density lipoprotein cholesterol, PIV = pan-immune-inflammation value, SII = systemic immune inflammation index, SIRI = system inflammation response index, TC = total cholesterol, TG = triglyceride.

* To prevent multicollinearity, analysis was carried out separately with these parameters.

### 3.3. Bivariate correlations

**Figure 4.**
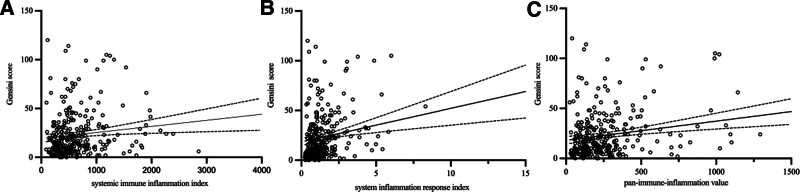
In ACS patients, the scatter plot illustrates the relationship between the SII, SIRI, or PIV and Gensini score. It had a positive linear association. ACS = acute coronary syndrome, PIV = pan-immune-inflammation value, SII = systemic immune inflammation index, SIRI = system inflammation response index.

In ACS patients, the scatter plot in Figure 4 illustrates the relationship between the SII, SIRI, or PIV and Gensini score. It had a positive linear association. Inflammatory markers (SII, SIRI, and PIV) were correlated with the Gensini score (*R* = 0.143, *P* = .011, *R* = 0.190, *P* = .001, and *R* = 0.207, *P* < .001, respectively) (Table 5).

**Table 5:** Bivariate correlations between inflammatory markers and Gensini score.

Variable	*r*	*P*
SII	0.143	.011
SIRI	0.190	.001
PIV	0.207	<.001

Abbreviations: PIV = pan-immune-inflammation value, SII = systemic immune inflammation index, SIRI = system inflammation response index.

### 3.4. Predictive value of SII, SIRI, and PIV for NCL progression

**Figure 5.**
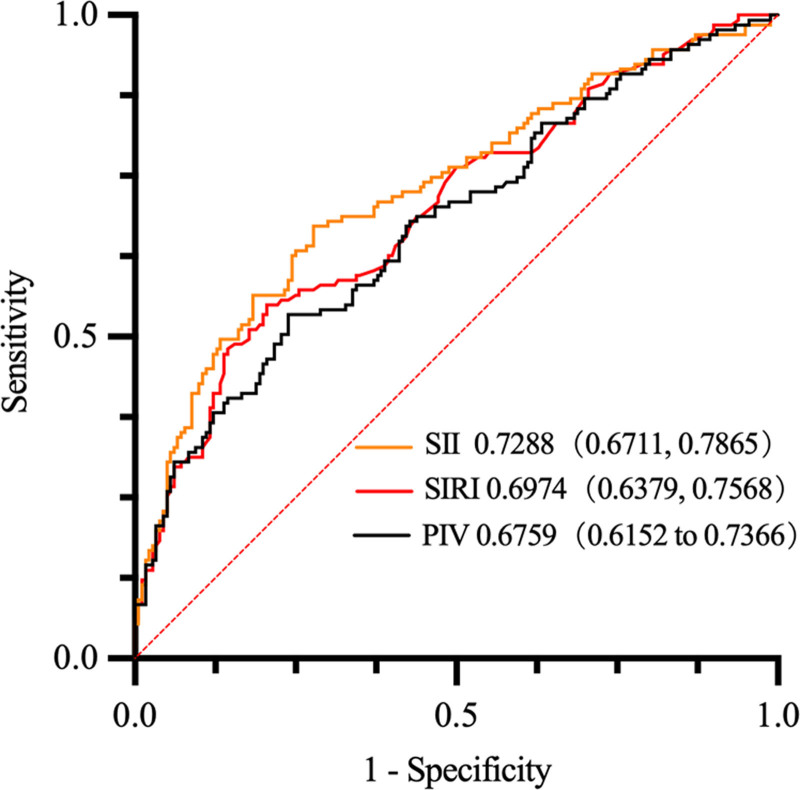
ROC curve of SII, SIRI, and PIV of NCL progression. The area under curve of SII is 0.7288, 95 CI: 0.6711, 0.7865. The area under curve of SIRI is 0.6974, 95 CI: 0.6974, 0.7568; The area under curve of PIV is 0.6759, 95 CI: 0.6152, 0.7366. NCL = non-culprit coronary lesion, PIV = pan-immune-inflammation value, ROC = receiver operating characteristic, SII = systemic immune inflammation index, SIRI = system inflammation response index.

ROC analysis was conducted for the progressive group patients, it showed that, with a cutoff value of >543.4 for SII, it predicted the NCL progression with 67.18% sensitivity and 72.22% specificity (AUC = 0.7288 [95% CI: 0.6711–0.7865], *P* < .001). For SIRI, a cutoff value of 1.355 predicted the NCL progression with a sensitivity of 54.96% and a specificity of 79.44% (AUC = 0.6974 [95% CI: 0.6379–0.7568], *P* < .001). For PIV, a cutoff value of 250.6 had 53.44% sensitivity and 76.11% specificity in predicting the NCL progression in patients with ACS (AUC = 0.6759 [95% CI: 0.6152–0.7366], *P* < .001) (Table 6, Fig. 5).

**Table 6:** ROC curve for NCL progression.

Variable	AUC	*P*	Cut off	Sensitivity	Specificity
SII	0.7288	<.0001	543.4	67.18%	72.22%
SIRI	0.6974	<.0001	1.355	54.96%	79.44%
PIV	0.6759	<.0001	250.6	53.44%	76.11%

Abbreviations: PIV = pan-immune-inflammation value, SII = systemic immune inflammation index, SIRI = system inflammation response index.

## 4. Discussion

We examined the levels of SII, SIRI, and PIV in the NCL progression group and the NCL non-progression group. Our findings indicated that SII, SIRI, and PIV levels in the NCL progression group are significantly higher than those in patients in the NCL non-progression group. Further investigation suggests that SII is the best in predicting the degree of vascular stenosis in NCL progression.

At present, the research on the progress mechanism of NCL mostly focuses on inflammatory reaction,^[9]^ lipogenic theory,^[3]^ oxidative stress theory,^[18]^ and hyper-homocysteinemia theory.^[19]^ Compared with other studies, this study mainly discusses the relationship between 3 new immune-inflammatory markers in the progress of NCL and adopts a cross-sectional study. Most existing studies adopt prospective studies, and the endpoints are readmission, re-PCI treatment, MACE incidence, or mortality. This study can preliminarily evaluate the progress of NCL after PCI through inflammatory markers, providing strong evidence for early intervention by clinicians.

Many patients with recurrent MACE after PCI was not associated with implanted stents but were attributed to the appearance of NCL progression and vulnerable plaques.^[20,21]^ Atherosclerosis is still the pathological basis of the progression of NCL, and its rapid plaque progression has become 1 of the primary problems facing patients after PCI.^[22]^ Although using lipid-lowering drugs such as statins in clinics is beneficial in delaying the progress of atherosclerotic plaque, it is difficult to stop the progress of plaque lesions. Cutlip et al^[20]^ pointed out that after 1 year of standardized treatment for low-risk people, the annual average risk rate of NCL accidents was 1.7%, and the annual average risk rate of NCL accidents was 6.3%, which was significantly higher than the incidence of CL. Varenhorst et al^[21]^ found that the risk of recurrent myocardial infarction in untreated NCL was twice as high as in previous stent treatment. Therefore, the progress of NCL is closely related to the recurrence of cardiovascular adverse events, which seriously affects the long-term prognosis of patients after PCI.

New immune-inflammatory markers can quantify some immune and inflammatory components in the body, including several inflammatory indicators that are easy to obtain and cheap.^[23]^ Neutrophils are one of the important subtypes of white blood cells, which are closely related to inflammatory reactions and can accelerate all stages of atherosclerosis.^[24]^ It is mainly by promoting monocyte recruitment, macrophage activation, and cytotoxicity.^[10]^ Monocytes can migrate and transform into macrophages when inflammation occurs, and secrete enzymes to promote the instability of fibrous cap and participate in plaque rupture.^[12,25]^ Many interventions have been proven to protect myocardial function after acute myocardial infarction by promoting the transition of monocytes/macrophages from pro-inflammatory phenotype to anti-inflammatory phenotype.^[26,27]^ Lymphocytes play an important role in developing atherosclerotic plaques, and lymphocytes participate in thrombosis when plaques rupture.^[28]^ The increase in platelet level may be related to the development and progress of CHD.^[29–31]^ As the cornerstone of the prevention and treatment of acute cardiovascular events, the benefits of antiplatelet drugs mainly come from their anti-aggregation effect and anti-inflammatory off-target effect.^[14]^ Previous studies have shown lower lymphocyte count is associated with higher cardiovascular disease risk.^[32]^ The higher neutrophil to lymphocyte ratio at admission also indicates that patients have a higher inflammatory response. Avci et al^[33]^ found that neutrophil to lymphocyte ratio was a valuable index in predicting the risk of hospital death of ST segment elevation myocardial infarction patients. Based on this, it is necessary to study the relationship between the new inflammatory indicators composed of the above indicators and the progress of NCL.

Many studies have confirmed that SII is a valuable predictor, which can predict the development trend of coronary artery disease,^[34]^ no-reflow phenomenon of ACS patients,^[35]^ the occurrence of preeclampsia in pregnant women,^[36]^ but also evaluate the load of thrombus.^[37]^ SIRI was associated with the diagnosis and had the highest values for patients with ACS.^[38]^ For acute type A aortic dissection, SIRI is significantly associated with the short-term and long-term prognosis of patients who underwent emergency. Sen et al^[39]^ showed that blood flow damage after PCI was related to high PIV before operation. In addition, Kaplangoray et al^[40]^ found that PIV is related to slow coronary blood flow. This shows that SII, SIRI, and PIV can be used as indicators to identify the risk of cardiovascular disease in individuals.

In this study, the data of 311 patients with ACS after PCI were analyzed. The interval between PCI was about 1.05 years, and the progress of NCL was observed in 42.1% of patients. First of all, our results show that the levels of SII, SIRI, and PIV in patients in the NCL progression group are significantly higher than those in patients in the NCL non-progression group. Secondly, when we compare the progress rate of NCL, we find that higher levels of SII, SIRI, and PIV are related to the increase in NCL progress rate. Secondly, when comparing the progress rate of NCL, we found that higher levels of SII, SIRI, and PIV were related to the increase in NCL progress rate. In addition, our analysis shows that after multivariate Logistic regression, SII, SIRI, and PIV are still independent predictors of NCL progress. This suggests that immune-inflammatory indexes (SII, SIRI, and PIV) can promote the progress of NCL in ACS patients after PCI. Finally, we compared the predictive efficacy of SII, SIRI, and PIV in predicting the degree of coronary artery stenosis in patients with NCL progression after PCI, and found that SII had the highest AUC and the best predictive efficiency. In addition, through bivariate correlation, SII, SIRI, and PIV were positively correlated with the Gensini score, among which PIV had the strongest correlation.

Gensini score is a comprehensive score to evaluate the angiographic load and severity of CHD patients. It is reported that the Gensini score is closely related to the atherosclerotic plaque load assessed by intravascular ultrasound.^[41]^ Therefore, the evaluation of NCL plaque in this study is reasonable. It can be seen that in ACS patients with elevated SII, SIRI, and PIV, the higher the Gensini score, the more severe the corresponding coronary stenosis and the more severe the plaque load.

Our research shows that SII is more closely related to the progress of NCL than other markers. This is different from the existing research point of view. At present, it is generally believed that the influence of more parameters (such as PIV with 4 parameters) can increase the value of biomarkers,^[42,43]^ but our research has not found it. Perhaps this is related to PIV and long-term prognosis.

Smoking and blood lipid-related indicators are independent risk factors for atherosclerosis, which can lead to the progression of NCL by regulating inflammation and immune response, which is consistent with previous studies.^[3,44]^ Additionally, diabetes mellitus is an independent risk factor for coronary artery disease. Berry et al^[45]^ found that FBG, HbA1c, and diabetes history were related to the severity and progress of coronary atherosclerosis. Wang et al^[46]^ conducted a follow-up study involving 205 patients with acute myocardial infarction concluding that FBG serves as an independent predictor of NCL progress in ST segment elevation myocardial infarction patients after direct PCI, our study also found that FBG and diabetes mellitus history were in NCL in CHD patients after PCI.

Although ACS is mainly caused by the rupture or erosion of criminal plaques, we should also pay attention to the progress of noncriminal vascular plaques. In this study, we compare the performance of SII, SIRI, and PIV, and find that the AUC of SII is the best in predicting the progress of NCL. For ACS patients treated by PCI, we can simply calculate the values of SII, SIRI, and PIV to evaluate the progress of patients’ noncriminal blood vessels. For patients with higher SII, we can have more reasons to check optical coherence tomographythrough PCI to determine the situation of NCL plaque and effectively prevent the occurrence of acute adverse cardiovascular events. Consistent with these findings, our study also demonstrates that FBG and a history of diabetes mellitus are significant factors in the presence of NCL among patients with ACS.

## 5. Research limitations

This study has several limitations. First, this study is a single-center retrospective analysis with a relatively small sample size, requiring further randomized prospective controlled study. Secondly, the lack of detailed vascular imaging data prevents a comprehensive assessment of the plaque. Third, blood samples were collected at the time of patient admission without dynamic observation of clinical outcomes. Fourth, the relationship between inflammatory markers of different types of ACS for the progression of NCL in patients was not further evaluated.

## 6. Conclusion

SII, SIRI, and PIV served as independent predictors of NCL progression, and the predictive value of SII was moderate. The Higher SII, SIRI, and PIV are Related to the Progression of NCL.

## Author contributions

**Data curation:** Zhou Yilu, Wang Zhanglong, Huang Fanke, Guan Jing, Wang Yue, Chen Yuwen, Li Bingqing.

**Formal analysis:** Wang Zhanglong, Zhou Yilu.

**Methodology:** Zhou Yilu

**Visualization:** Zhou Yilu.

**Writing – original draft:** Zhou Yilu.

**Writing – review & editing:** Yilu Zhou, Lv Jianfeng.

## References

[1] Vaduganathan M Mensah GA Turco JV Fuster V Roth GA. The global burden of cardiovascular diseases and risk: a compass for future health. J Am Coll Cardiol. 2022;80:2361–71. 36368511 10.1016/j.jacc.2022.11.005

[2] Libby P Theroux P. Pathophysiology of coronary artery disease. Circulation. 2005;111:3481–8. 15983262 10.1161/CIRCULATIONAHA.105.537878

[3] Koskinas KC Mach F Raber L. Lipid-lowering therapy and percutaneous coronary interventions. EuroIntervention. 2021;16:1389–403. 33875408 10.4244/EIJ-D-20-00999 PMC9890584

[4] Mehta SR Wood DA Storey RF; COMPLETE Trial Steering Committee and Investigators. Complete revascularization with multivessel PCI for myocardial infarction. N Engl J Med. 2019;381:1411–21. 31475795 10.1056/NEJMoa1907775

[5] Zimbarod G Cialdella P Di FP. Acute coronary syndromes and multivessel coronary artery disease. Eur Heart J Suppl. 2023;25(Suppl C):C74–C8. 37125291 10.1093/eurheartjsupp/suad010 PMC10132620

[6] Asakura M Ueda Y Yamaguchi O. Extensive development of vulnerable plaques as a pan-coronary process in patients with myocardial infarction: an angioscopic study. J Am Coll Cardiol. 2001;37:1284–8. 11300436 10.1016/s0735-1097(01)01135-4

[7] Wang X Xie Z Liu X. Association of Platelet to lymphocyte ratio with non-culprit atherosclerotic plaque vulnerability in patients with acute coronary syndrome: an optical coherence tomography study. BMC Cardiovasc Disord. 2017;17:175. 28673240 10.1186/s12872-017-0618-y PMC5496410

[8] Baena-Diez JM Garcia-Gil M Comas-Cufi M. Association between chronic immune-mediated inflammatory diseases and cardiovascular risk. Heart. 2018;104:119–26. 28847852 10.1136/heartjnl-2017-311279

[9] Mehu M Narasimhulu CA Singla DK. Inflammatory cells in atherosclerosis. Antioxidants (Basel). 2022;11:233. 35204116 10.3390/antiox11020233 PMC8868126

[10] Silvestre-Roig C Braster Q Ortega-Gomez A Soehnlein O. Neutrophils as regulators of cardiovascular inflammation. Nat Rev Cardiol. 2020;17:327–40. 31996800 10.1038/s41569-019-0326-7

[11] Weber C Noels H. Atherosclerosis: current pathogenesis and therapeutic options. Nat Med. 2011;17:1410–22. 22064431 10.1038/nm.2538

[12] Ruder AV Wetzels SMW Temmerman L Biessen EAL Goossens P. Monocyte heterogeneity in cardiovascular disease. Cardiovasc Res. 2023;119:2033–45. 37161473 10.1093/cvr/cvad069 PMC10478755

[13] Nunez J Minana G Bodi V. Low lymphocyte count and cardiovascular diseases. Curr Med Chem. 2011;18:3226–33. 21671854 10.2174/092986711796391633

[14] Muller KA Chatterjee M Rath D Geisler T. Platelets, inflammation and anti-inflammatory effects of antiplatelet drugs in ACS and CAD. Thromb Haemost. 2015;114:498–518. 26224127 10.1160/TH14-11-0947

[15] Zheng JL Lu L Hu J. Increased serum YKL-40 and C-reactive protein levels are associated with angiographic lesion progression in patients with coronary artery disease. Atherosclerosis. 2010;210:590–5. 20056225 10.1016/j.atherosclerosis.2009.12.016

[16] Fuca G Guarini V Antoniotti C. The pan-immune-inflammation value is a new prognostic biomarker in metastatic colorectal cancer: results from a pooled-analysis of the Valentino and TRIBE first-line trials. Br J Cancer. 2020;123:403–9. 32424148 10.1038/s41416-020-0894-7 PMC7403416

[17] Wang KY Zheng YY Wu TT Ma Y-T Xie X. Predictive value of gensini score in the long-term outcomes of patients with coronary artery disease who underwent PCI. Front Cardiovasc Med. 2021;8:778615. 35141291 10.3389/fcvm.2021.778615 PMC8818732

[18] Buffon A Santini SA Ramazzotti V. Large, sustained cardiac lipid peroxidation and reduced antioxidant capacity in the coronary circulation after brief episodes of myocardial ischemia. J Am Coll Cardiol. 2000;35:633–9. 10716465 10.1016/s0735-1097(99)00581-1

[19] Jan M Cueto R Jiang X. Molecular processes mediating hyperhomocysteinemia-induced metabolic reprogramming, redox regulation and growth inhibition in endothelial cells. Redox Biol. 2021;45:102018. 34140262 10.1016/j.redox.2021.102018 PMC8282538

[20] Cutlip DE Chhabra AG Baim DS. Beyond restenosis: five-year clinical outcomes from second-generation coronary stent trials. Circulation. 2004;110:1226–30. 15337693 10.1161/01.CIR.0000140721.27004.4B

[21] Varenhorst C Hasvold P Johansson S. Culprit and nonculprit recurrent ischemic events in patients with myocardial infarction: data from SWEDEHEART (Swedish Web System for Enhancement and Development of Evidence-Based Care in Heart Disease Evaluated According to Recommended Therapies). J Am Heart Assoc. 2018;7:e007174. 31913732 10.1161/JAHA.117.007174 PMC5778965

[22] Scirica BM Bergmark BA Morrow DA. Nonculprit lesion myocardial infarction following percutaneous coronary intervention in patients with acute coronary syndrome. J Am Coll Cardiol. 2020;75:1095–106. 32164882 10.1016/j.jacc.2019.12.067

[23] Xia Y Xia C Wu L Li Z Li H Zhang J. Systemic Immune Inflammation Index (SII), System Inflammation Response Index (SIRI) and risk of all-cause mortality and cardiovascular mortality: a 20-year follow-up cohort study of 42,875 US adults. J Clin Med. 2023;12:1128. 36769776 10.3390/jcm12031128 PMC9918056

[24] Avci B Avci A Donmez Y. The effectiveness of neutrophil-lymphocyte ratio in predicting in-hospital mortality in non-ST-elevation myocardial infarction. Emerg Med Int. 2020;2020:8718304. 32211208 10.1155/2020/8718304 PMC7085368

[25] Pasterkamp G Van Keulen JK De Kleijn DP. Role of Toll-like receptor 4 in the initiation and progression of atherosclerotic disease. Eur J Clin Invest. 2004;34:328–34. 15147329 10.1111/j.1365-2362.2004.01338.x

[26] Tian Y Piras BA Kron IL French BA Yang Z. Adenosine 2B receptor activation reduces myocardial reperfusion injury by promoting anti-inflammatory macrophages differentiation via PI3K/Akt pathway. Oxid Med Cell Longev. 2015;2015:585297. 26161239 10.1155/2015/585297 PMC4486757

[27] Kim Y Nurakhayev S Nurkesh A. Macrophage polarization in cardiac tissue repair following myocardial infarction. Int J Mol Sci. 2021;22:2715. 33800220 10.3390/ijms22052715 PMC7962533

[28] Abdolmaleki F Gheibi HSM Bianconi V. Atherosclerosis and immunity: a perspective. Trends Cardiovasc Med. 2019;29:363–71. 30292470 10.1016/j.tcm.2018.09.017

[29] Ibrahim H Schutt RC Hannawi B DeLao T Barker CM Kleiman NS. Association of immature platelets with adverse cardiovascular outcomes. J Am Coll Cardiol. 2014;64:2122–9. 25457402 10.1016/j.jacc.2014.06.1210

[30] Aadre P Nannizzi-Alaimo L Prasad SK. Platelet-derived CD40L: the switch-hitting player of cardiovascular disease. Circulation. 2002;106:896–9. 12186789 10.1161/01.cir.0000028962.04520.01

[31] Chen WA Boskovic DS. Neutrophil extracellular DNA traps in response to infection or inflammation, and the roles of platelet interactions. Int J Mol Sci. 2024;25:3025. 38474270 10.3390/ijms25053025 PMC10932463

[32] Nunez J Sanchis J Bodi V. Therapeutic implications of low lymphocyte count in non-ST segment elevation acute coronary syndromes. Eur J Intern Med. 2009;20:768–74. 19892306 10.1016/j.ejim.2009.09.006

[33] Avci A Avci BS Donmez Y. Which one predicts mortality better? Hemogram and ST elevation myocardial infarction. Niger J Clin Pract. 2019;22:598–602. 31089012 10.4103/njcp.njcp_540_18

[34] Yang YL Wu CH Hsu PF. Systemic immune-inflammation index (SII) predicted clinical outcome in patients with coronary artery disease. Eur J Clin Invest. 2020;50:e13230. 32291748 10.1111/eci.13230

[35] Ndrepepa G Tiroch K Keta D. Predictive factors and impact of no reflow after primary percutaneous coronary intervention in patients with acute myocardial infarction. Circ Cardiovasc Interv. 2010;3:27–33. 20118156 10.1161/CIRCINTERVENTIONS.109.896225

[36] Kapci M Sener K Cakir A Altug E Guven R Avci A. Prognostic value of systemic immune-inflammation index in the diagnosis of preeclampsia. Heliyon. 2024;10:e28181. 38560698 10.1016/j.heliyon.2024.e28181 PMC10979240

[37] Dai F Xu X Zhou C. Development and validation of a nomogram to predict the five-year risk of revascularization for non-culprit lesion progression in STEMI patients after primary PCI. Front Cardiovasc Med. 2023;10:1275710. 38094123 10.3389/fcvm.2023.1275710 PMC10716459

[38] Dziedzic EA Gasior JS Tuzimek A. Investigation of the associations of novel inflammatory biomarkers-systemic inflammatory index (SII) and systemic inflammatory response index (SIRI)-with the severity of coronary artery disease and acute coronary syndrome occurrence. Int J Mol Sci. 2022;23:9553. 36076952 10.3390/ijms23179553 PMC9455822

[39] Şen F Kurtul A Bekler O. Pan-Immune-inflammation value is independently correlated to impaired coronary flow after primary percutaneous coronary intervention in patients with ST-segment elevation myocardial infarction. Am J Cardiol. 2024;211:153–9. 37944774 10.1016/j.amjcard.2023.10.088

[40] Kaplangoray M Toprak K Deveci E Caglayan C Şahin E. Could pan-immune-inflammation value be a marker for the diagnosis of coronary slow flow phenomenon? Cardiovasc Toxicol. 2024;24:519–26. 38622332 10.1007/s12012-024-09855-4 PMC11076385

[41] Neeland IJ Patel RS Eshtehardi P. Coronary angiographic scoring systems: an evaluation of their equivalence and validity. Am Heart J. 2012;164:547–52.e1. 23067913 10.1016/j.ahj.2012.07.007 PMC3913177

[42] Murat B Murat S Ozgeyik M Bilgin M. Comparison of pan-immune-inflammation value with other inflammation markers of long-term survival after ST-segment elevation myocardial infarction. Eur J Clin Invest. 2023;53:e13872. 36097823 10.1111/eci.13872

[43] BayramogluA A Hidayet S. Association between pan-immune-inflammation value and no-reflow in patients with ST elevation myocardial infarction undergoing percutaneous coronary intervention. Scand J Clin Lab Invest. 2023;83:384–9. 37498164 10.1080/00365513.2023.2241131

[44] Buljubasic N Akkerhuis KM De Boer SP. Smoking in relation to coronary atherosclerotic plaque burden, volume and composition on intravascular ultrasound. PLoS One. 2015;10:e0141093. 26491969 10.1371/journal.pone.0141093 PMC4619630

[45] Berry C Noble S Gregoire JC. Glycaemic status influences the nature and severity of coronary artery disease. Diabetologia. 2010;53:652–8. 20225394 10.1007/s00125-009-1651-x

[46] Wang J Yan CY Wang W Wang T-Z. The clinical prediction factors for non-culprit lesion progression in patients with acute ST elevation myocardial infarction after primary percutaneous coronary intervention. BMC Cardiovasc Disord. 2022;22:529. 36474153 10.1186/s12872-022-02974-2 PMC9724424

